# Causal relationship between immune cells and myocardial infarction: A Mendelian randomization study

**DOI:** 10.1097/MD.0000000000043682

**Published:** 2025-09-12

**Authors:** Yinyin Xu, Jing Yang, Rong Xue, Guojiang Zhang, Yanhua Zhang

**Affiliations:** a Department of Cardiology, Third People's Hospital of Datong, Datong, Shanxi Province, China; b The First Clinical Medical College of Changzhi Medical College, Changzhi, Shanxi Province, China.

**Keywords:** causal relationship, GWAS, immune cells, Mendelian randomization study, myocardial infarction

## Abstract

Myocardial infarction (MI) is a major cause of death worldwide. It is been suspected for a long time that MI is linked to immune cells. However, observational studies are plagued by confounding factors and reverse causality, whether the immune response is a cause or consequence of MI remains unknown. The present study aimed to determine whether genetically immune cells might have a causal effect on MI. According to publicly available genetic data, we assessed the causal relationship between 731 immune cell signatures (7 groups) and MI based on a two-sample Mendelian randomization (MR) analysis. Single nucleotide polymorphisms from a genome-wide association study comprising 3757 Sardinians on immune cells were used as exposure instruments. Another summary-level genome-wide association study statistics of MI were used as the outcome data. We primarily used inverse variance weighted, MR-egger, and simple median methods to perform MR analyses. Comprehensive sensitivity analyses were used to verify the robustness, heterogeneity, and horizontal pleiotropy of the results. Twenty-seven of 731 immune cell phenotypes are causally associated with MI (odds ratio: 0.94–1.06, 95% confidence interval: 0.80–1.15, *P* < .048). Among them, 14 immunophenotypes were negatively associated with the occurrence of MI, in other words, the more these immune cell phenotypes, the lower the probability of MI. The remaining 13 immunophenotypes were positively correlated with MI. Our study has demonstrated the close connection between immune cells and MI by genetic means, and revealed the direct causal relationship between these immune cells and MI with the help of MR experiments, which to a certain extent avoids the wastage of manpower, resources, and finance that would be incurred by opening up a large-scale clinical trial to obtain unsatisfactory results. On the other hand, these immune cells shown in our results may become new biomarkers of MI or even potential drug targets for the treatment of MI, thus providing a new target for prevention, diagnosis, and treatment of MI.

## 1. Introduction

Myocardial infarction (MI) is one of the life-threatening coronary events that leads to sudden cardiac death and is the most serious clinical manifestation of coronary artery disease (CAD). A published study found that the prevalence of MI was 27.0% in 19,781 patients with CAD.^[1]^ While heart attack morbidity and mortality have declined in recent years as a result of the increased use of evidence-based medications and percutaneous coronary intervention, studies have shown that the incidence of heart attacks has not declined as much in younger people as in older people.^[2,3]^ And MI is firmly at the top of the list of leading causes of death globally, making it the leading cause of death in industrialized countries.^[4,5]^ A prognostic study showed that the incidence of MI is projected to jump 194.4% from 2025 to 2050, from 482 to 1418 cases per 100,000 population.^[6]^ Worldwide spending on cardiovascular disease hospitalizations, therapies, revascularization procedures, outpatient, emergency, and prescription drug treatments is expected to exceed $1 trillion by 2030, according to the World Heart Federation.^[7]^ All these studies and their data suggest that further exploration of the pathogenesis of MI and finding ways to prevent and treat MI are essential.

The primary pathological process that leads to CAD is atherosclerosis, an inflammatory disease of the arteries associated with lipid deposition and metabolic alterations due to multiple risk factors.^[7]^ The coronary atherosclerosis is a chronic disease with periods of stability and instability. During the unstable period of inflammatory activation of the vessel wall, patients may develop MI.^[8]^ Distinct inflammatory participants play pivotal pathological functions in (i) driving atherosclerosis during the clinically stable stage of the disease, (ii) triggering plaque destabilization and so evoking acute coronary syndromes (ACS), and (iii) responding to cardiomyocyte death in MI.^[9]^ A variety of immune cells play an important role in the occurrence and development of MI, some of which cause atherosclerosis, MI, and heart failure, while others prevent the corresponding lesions. For instance, macrophages are involved in both damage and repair responses during MI.^[10]^ Leo M Carlin and Quintar et al demonstrated the protective role of patrolling monocytes in maintaining vascular homeostasis.^[11,12]^ From the other hand, Urbanski et al noted that in patients with CAD, high levels of non-classical monocytes in the blood were associated with more aggressive vascular dysfunction and vascular oxidative stress.^[13]^ Biomarkers of inflammation have been proven to be predictive of MI, independently of conventional risk factors.^[14]^ The inflammatory biology of atherosclerosis and MI has been translated into therapeutic tactics over the past few years, but the identification of effective management is not yet fully understood.^[14]^ Multiple trials that addressed the early inflammatory response after MI yielded a neutral result. These failures may be in part due to underrecognition of the inter-individual variability of the inflammatory response, which may be motivated by differential environmental or genetic predisposition.^[9]^

MI is still the leading cause of death worldwide. Therefore, it is of interest to study the causes and mechanisms of MI. While randomized trials are in principle the best way of determining the causal status of a particular exposure, they have some limitations. For example, they are expensive, time consuming and impractical, even immoral. What’s more, confusion or reverse causality may provide a biased. More reliable approaches are therefore needed for causality assessment using observational data. Mendelian randomization (MR) is one such approach. The random assignment of individuals to receive exposure was simulated by selecting an instrumental variable, thus ensuring comparability between groups under any known and unknown confounders. When such a validated tool is available, the effect of exposure on outcome can be estimated without bias, allowing for the assessment of causality of observed associations.^[15]^ Although instruments can be used for essentially any variable, genetic variants, such as single nucleotide polymorphisms (SNPs), are increasingly being used because during human gametogenesis, alleles of a given SNP are randomly distributed into the oocyte/sperm cell, that is, alleles of the SNP are assigned to an individual prior to any exposure or outcome. Thus, genetic variation is independent of the potential confounding environment.^[16]^

In this study, a comprehensive two-sample MR analysis was performed to determine the causal relationship between immune cell characteristics and MI, and further screen out immune cells related to MI occurrence to provide clues for preventive measures and treatment strategies for MI.

## 2. Materials and methods

### 2.1. Study design

We assessed the causal relationship between 731 immune cell signatures (7 groups) and MI based on a two-sample MR analysis. The selection of a genetic instrumental variable (GIV) is critical to the success of MR studies. In order to be able to estimate exposure without bias effect on the result of cause and effect, effective GIV to satisfy 3 core assumptions: (1) it must be reproducible and strongly associated with exposure. (2) It must not be related to confounders (i.e., factors that confound the relationship between exposure and outcome). (3) It is only related to the outcome of exposure (that is, it is not related to the outcome of exposure). All study procedures were conducted in accordance with the World Medical Association Helsinki Declaration of Ethical Principles for Medical Research. Ethical approval was not considered to be required for this study because the included genome-wide association study (GWAS) reported appropriate ethical approval from their respective institutions and this analysis was only performed on abstract-level data.

### 2.2. GWAS data sources for MI

GWAS summary statistics for MI were obtained from the UK Biobank and CARDIoGRAMplusC4D Consortium, the study included 14,825 cases and 44,000 controls of European ancestry (accession number GCST011365). Approximately 8106,745 single SNPs were tested.^[17]^

### 2.3. Immunity-wide GWAS data sources

GWAS summary statistics for each immune trait are publicly available from the GWAS Catalog (accession numbers from GCST90001391–GCST90002121). These data were derived from a genome-wide association study that enriched genetic variation in the genetic components of 731 immunophenotypes previously identified by genome sequencing.^[18]^ Cellular phenotypes were analyzed by antibody staining and flow cytometry, and donor peripheral blood was classified into 7 groups: B cells, dendritic cells (DCs), T-cell maturation stage, monocytes, myeloid cells, TBNK (T cells, B cells, natural killer cells), and regulatory T cell (Treg) panels, including absolute cell counts (n = 118), median fluorescence intensities reflecting surface antigen levels (n = 389), morphological parameters (n = 32) and relative cell counts (n = 192).^[19]^

### 2.4. Selection of instrumental variables

In order to meet the 3 assumptions of MR experiments, SNPs should generally be selected for genome-wide significance, which is defined as *P* < 5 × 10^−8^. However, when we screened immunophenotypes using this criterion, we were unable to find eligible SNPs, so we chose a lower *P*-value based on previously published literature.^[19,20]^ The significance level of instrumental variables for each immune trait was set to 1 × 10^-5^. With PLINK software (v1.90 version) of clumping program clip these SNPs (linkage disequilibrium *r*^2^ threshold < 0.1 within 500 kb distance), of which linkage disequilibrium *r*^2^ is calculated according to the 1000 Genomes Project as a reference group. The F statistic was calculated to measure the strength of the tools. All genetic tools had F-values >10 to ensure that at least 95% of the time the weak bias was <10.^[21]^

### 2.5. Statistical analyses

All MR analyses and visualizations were conducted using TwoSampleMR package in R software 4.3.2. Binary end effect estimate report for odds ratio and corresponding 95% confidence interval (CI).

To assess the causal relationship between the 731 immunophenotypes and MI, inverse variance weighting (IVW) was used as the primary analysis, with the use of “MendelianRandomization” package, version 0.4.26. We then applied a series of sensitivity analyses to assess the robustness of the IVW results to potential violations, including MR-Egger, simple median, and MR-PRESSO. While these methods themselves have relatively low statistical efficiency, they have different theoretical properties to control for different types of bias, and they are robust to certain assumption violations.

The IVW approach (fixed-effects model) provided the greatest power, assuming that all genetic instruments were valid.^[22]^ This method is equivalent to a weighted linear regression of the SNP exposure effect on the SNP outcome effect with an intercept restricted to zero. Because of this limitation, it may lead to relatively high false-positive rates in the presence of horizontal pleiotropy. Cochran Q statistic from IVW analysis was used for global heterogeneity testing.^[23]^ Based on the concept that pleiotropy is one of the main sources of heterogeneity, low heterogeneity (Cochran Q *P* > .05) means that pleiotropy is less likely.

The MR-Egger regression was performed similarly to IVW, but the intercept was not fixed to zero.^[24]^ Thus, even in the presence of pleiotropy, the slope coefficient of the MR-Egger regression can provide an adjusted causal estimate. The intercept of the MR-Egger regression is an indicator of the genetic pleiotropy effect between genetic variants. A zero intercept associated with *P* > .05 was considered to be evidence for the absence of pleiotropic bias. Even if all selected SNPs are invalid, the MR-Egger method can also provide unbiased estimation.^[24]^

The simple median method, which provides effect estimates even when 50% of the genetic instrument are invalid.^[25]^

The MR-PRESSO is an extension of the IVW method. The MR-PRESSO global test was used to assess the presence of overall level pleiotropy. If pleiotropy is detected, the MR-PRESSO outlier test allows the detection of a single pleiotropy outlier by calculating the sum of squares of the residuals. Finally, causal estimates were obtained by applying the IVW method to the genetic variants remaining after exclusion of outliers.^[26]^

Leave-one-out (LOO) sensitivity analysis is a method finally used to determine whether the association, and the result of a single SNP driven by any single SNP.

**Figure 1.**
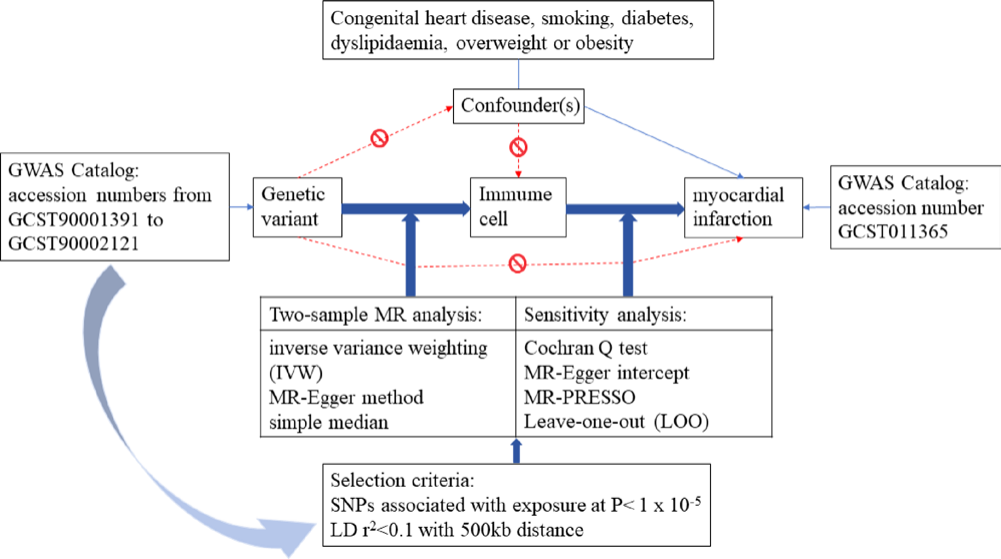
Three hypotheses and data analysis.

In addition, scatter plots and funnel plots were used. Scatter plots show that the results are not affected by outliers. The funnel plot shows the robustness of the association and the absence of heterogeneity. The flowchart of data sources and analysis is shown in Figure 1.

## 3. Results

In the absence of significant heterogeneity in the Cochran Q test (*P* > .05), a fixed-effects model was employed to estimate the effect size of MR. Through MR experiments, IVW in 731 kinds of immune phenotypes of detected 27 kinds possibly associated with MI, including 7 in B cell population, 1 in DCs population, 16 in TBNK population, 3 in monocytes population, and 1 in myeloid population. The cell panel to which the 27 immune cells belong are shown in Table 1. Among them, 13 immunophenotypes were negatively associated with the occurrence of MI, in other words, the more immune cells belonging to the immunophenotype, the less likely MI would occur. Including IgD + CD38 + B cell %lymphocyte (95% CI = 0.92–0.99, *P* = 6.9 × 10^−3^), Naive-mature B cell %lymphocyte (95% CI = 0.96–1.00, *P* = 4.8 × 10^−2^), effector memory CD4-CD8- T cell %T cell (95% CI = 0.96~1.00, *P* = 4.8 × 10^−2^), transitional B cell %B cell (95% CI = 0.92–1.00, *P* = 4.2 × 10^−2^), CD28 + CD45RA + CD8 + T cel %CD8 + T cell (95% CI = 0.99–1.00, *P* = 2.2 × 10^−2^), CD19 on lgD-CD24- B cell (95% CI = 0.93–0.99, *P* = 9.3 × 10^−3^), CD19 on unswitched memory B cell (95% CI = 0.91–1.00, *P* = 3.3 × 10^−2^), IgD on IgD + CD24- B cell (95% CI = 0.93–0.99, *P* = 5.5 × 10^−3^), CD45 on CD4 + T cell 95% CI = 0.91–0.98, *P* = 1.8 × 10^−3^), FSC-A on human leukocyte antigen (HLA) DR + natural killer (95% CI = 0.96–0.99, *P* = 8.0 × 10^−3^), CD40 on CD14 + CD16- monocyte (95% CI = 0.97–1.00, *P* = 2.0 × 10^−2^), CD45 on lymphocyte (95% CI = 0.96–1.00, *P* = 3.6 × 10^−2^), HLA DR on HLA DR + T cell (95% CI = 1.01, *P* = 4.2 × 10^−2^). The 2 sensitivity analyses we used, including the MR-egger method and the simple median method, yielded essentially similar results, demonstrating the robustness of the results. On the other hand, the other 14 kinds of immune phenotypes were positively correlated with MI, namely the immune cells have the potential to contribute to the occurrence of MI. HLA DR++ monocyte absolute count (95% CI = 1.01–1.07, *P* = 1.8 × 10^−2^), hematopoietic stem cell absolute count (95% CI = 1.00–1.04, *P* = 2.4 × 10^−2^), terminally differentiated CD4 + T cell %Count (95% CI = 1.00–1.07, *P* = 3.2 × 10 − 2), terminally differentiated CD8 + T cell %T cell (95% CI = 1.00–1.05, *P* = 3.8 × 10^−2^), CD8dim natural killer T absolute count (95% CI = 1.01–1.06, *P* = 1.6 × 10^−2^), B cell absolute count (95% CI = 1.00–1.06, *P* = 2.5 × 10^−2^), CD28 + CD45RA- CD8dim T cell absolute count (95% CI = 1.00–1.01, *P* = 9.4 × 10^−3^), CD127 on CD28 + CD45RA + CD8 + T cell (95% CI = 1.01–1.11, *P* = 2.7 × 10^−2^), FSC-A on granulocyte (95% CI = 1.00~1.07, *P* = 4.7 × 10^−2^), CX3CR1 on CD14- CD16 + monocyte (95% CI = 1.01–1.05, *P* = 1.5 × 10^−2^), CD8 on CD8 + T cell (95% CI = 1.00–1.07, *P* = 4.5 × 10^−2^), CD4 on CD39 + activated CD4 regulatory T cell (95% CI = 1.01–1.08, *P* = 1.3 × 10^−2^), CD45RA on naive CD4 + T cell (95% CI = 1.00–1.03, *P* = 4.4 × 10^−2^), CD8 on CD28- CD8 + T cell (95% CI = 1.00–1.07, *P* = 3.0 × 10^−2^) are included. The sensitivity analysis almost proved the robustness of the above results, except for CD127 on CD28 + CD45RA + CD8 + T cells and CD28-CD8 + T cells, which showed inconsistent results using the MR-Egger method with the IVW and simple median methods. This is due to the shortcomings of the MR-Egger method itself, due to the lack of statistical efficacy of the method, which is susceptible to outlying SNPs with a larger standard deviation than the other methods, and the possible inclusion of SNPs with no detectable pleiotropic effect in the study, which can cause bias, and therefore we dominated with the final effect of the main method used, that is, IVW.

**Table 1:** Cell panel for each immunophenotype.

GWAS Number		Trait	Trait type	Panel	N samples
GCST90001429	IgD + CD38+ %lymphocyte		Relative count	B cell	3754
GCST90001437	Naive-mature B cell %lymphocyte		Relative count	B cell	3754
GCST90001477	HLA DR++ monocyte AC		Absolute count	TBNK	3579
GCST90001514	HSC AC		Absolute count	Myeloid cell	1930
GCST90001546	TD CD4+ %CD4+		Relative count	Maturation stages of T cell	3427
GCST90001559	TD CD8br %T cell		Relative count	Maturation stages of T cell	3427
GCST90001571	EM DN (CD4-CD8-) %T cell		Relative count	Maturation stages of T cell	3427
GCST90001576	Transitional %B cell		Relative count	B cell	3755
GCST90001633	CD8dim NKT AC		Absolute count	TBNK	3652
GCST90001642	B cell AC		Absolute count	TBNK	3653
GCST90001669	CD28 + CD45RA- CD8dim AC		Absolute count	Treg	3408
GCST90001689	CD28 + CD45RA + CD8br % CD8br		Relative count	Treg	3440
GCST90001731	CD19 on IgD- CD24-		MFI	B cell	3753
GCST90001738	CD19 on unsw mem		MFI	B cell	3754
GCST90001821	IgD on IgD + CD24-		MFI	B cell	3754
GCST90001916	CD45 on CD4+		MFI	TBNK	3113
GCST90001929	CD127 on CD28 + CD45RA + CD8br		MFI	Treg	2920
GCST90001966	FSC-A on granulocyte		Morphological parameter	cDC	2850
GCST90001970	FSC-A on HLA DR + NK		Morphological parameter	TBNK	2971
GCST90001980	CD40 on CD14 + CD16- monocyte		MFI	Monocyte	3727
GCST90002012	CX3CR1 on CD14- CD16 + monocyte		MFI	Monocyte	3679
GCST90002041	CD45 on lymphocyte		MFI	Myeloid cell	1635
GCST90002058	CD8 on CD8br		MFI	TBNK	3113
GCST90002067	CD4 on CD39 + activated Treg		MFI	Treg	2920
GCST90002098	CD45RA on naive CD4+		MFI	Maturation stages of T cell	2910
GCST90002113	HLA DR on HLA DR + T cell		MFI	TBNK	3060
GCST90002120	CD8 on CD28- CD8br		MFI	Treg	2920

**Figure 2.**
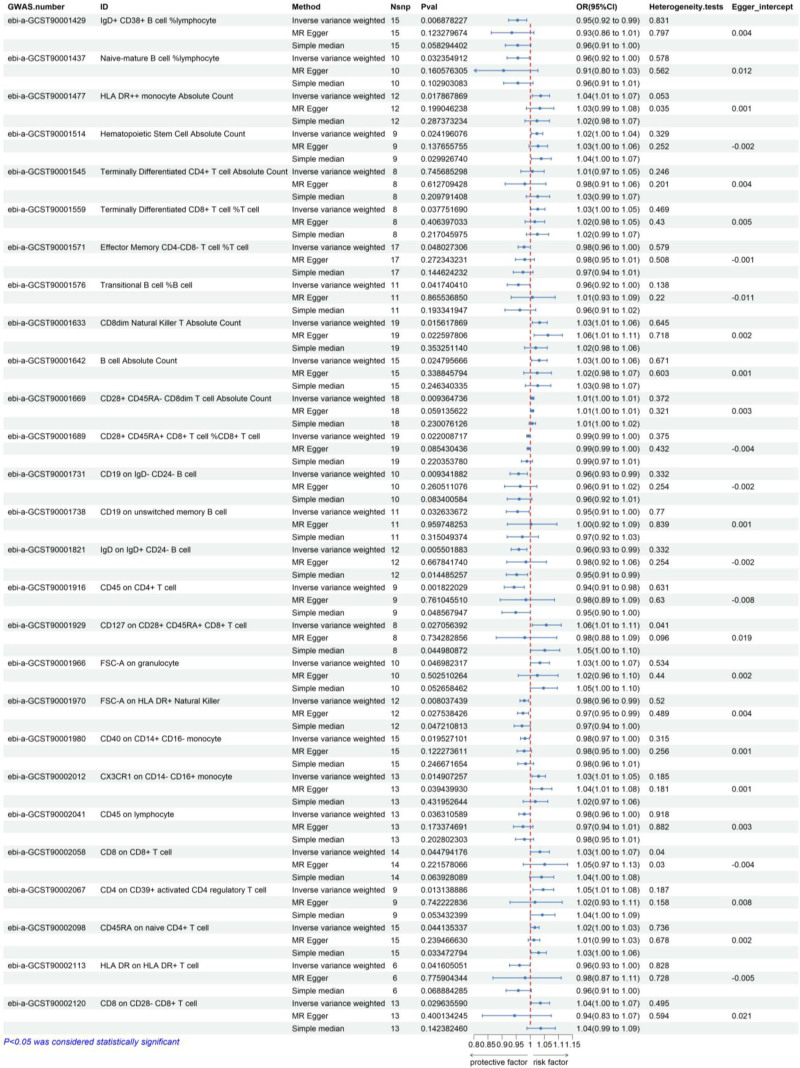
The forest plot of IVW method/MR-egger/simple median cue causality. IVW = inverse variance weighted, MR = Mendelian randomization.

**Figure 3.**
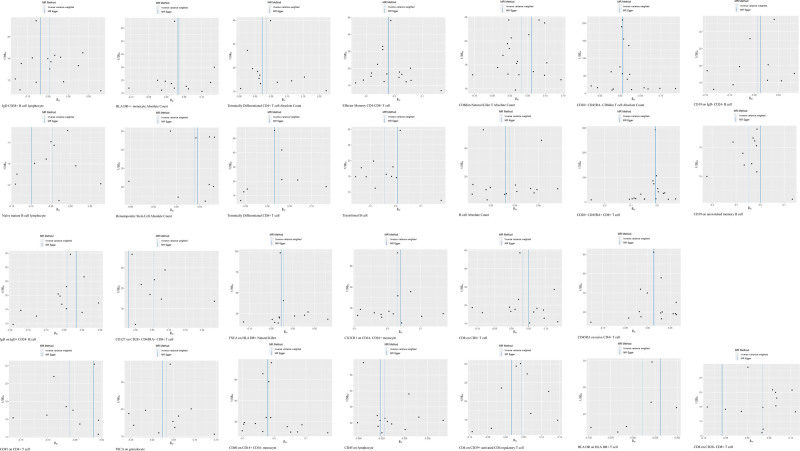
Funnel plots. Dots in the funnel plot represent SNPs included in the study. Vertical lines represent the combined ORs from the IVW and MR-Egger methods. IVW = inverse variance weighted, MR = Mendelian randomization, ORs = odds ratio, SNPs = single nucleotide polymorphisms.

**Figure 4.**
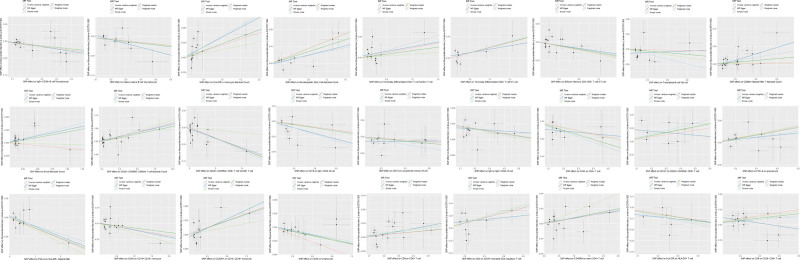
The scatterplot results showed that the regression lines derived from the various MR statistical methods were generally consistent. MR = Mendelian randomization.

**Figure 5.**
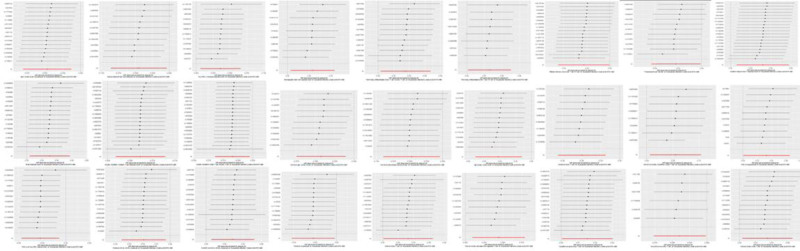
The LOO analysis further indicated that the significance of these results was not driven by any single SNP. LOO = leave-one-out, SNPs = single nucleotide polymorphisms.

Forest plots of the causal relationship between all immune cell characteristics and MI are shown in Figure 2. As shown in Figure 3, our funnel plots were all symmetrically distributed, suggesting that there was no heterogeneity in this study. Each point in the scatter plot represents a genetic variant SNP, and the different colored lines indicate different algorithms. as shown in Figure 4, our results show that the SNPs are uniformly dispersed around the lines, and the overall slopes of the lines for the different algorithms are the same, suggesting the consistency of the results and the robustness of the findings. Special cases such as CD127 on CD28 + CD45RA + CD8 + T cells and CD28-CD8 + T cells have been explained in the previous paragraph. LOO performed a culling analysis of the effect on the risk of MI for each immunophenotype. This approach was used to assess whether the combined IVW estimates were influenced by specific individual SNPs. If the MR results estimated after excluding a specific SNP were significantly different from the overall results, this would indicate that the MR results were more sensitive to the instrumental variables.The LOO analysis further indicated that the significance of these results was not driven by any single SNP. Detailed information is provided in Figure 5.

## 4. Discussion

On the basis of publicly available genetic data, we analyzed the causal relationship between multiple immune phenotypes and MI by MR experiments, and cellular categorization of 731 immune cell phenotypes. According to the above results, we can observe that all immune phenotypes in the B cell panel are positively associated with the occurrence of MI, and other immune phenotypes belonging to the TBNK panel, the Treg panel, the maturation stages of T-cell panel, and the DC panel are all favored a positive correlation with the occurrence of MI, while the immunophenotypes belonging to the monocyte panel and the myeloid cell panel had the same proportion of positive and negative effects on MI.

According to our study, 6 phenotypes belonging to the B cell panel, namely, IgD + CD38 + B cell lymphocyte, Naive − mature B cell lymphocyte, transitional B cell, CD19 on IgD − CD24 − B cell, CD19 on unswitched memory B cell, IgD on IgD + CD24 − B cell, prevented the development of MI. It is well known that the generation of vulnerable plaques is the initiating factor in the development of MI. Numerous studies have demonstrated that B cells are important regulators of atherosclerosis, vulnerable plaque formation, and MI. In preclinical models, B1 B cells are innate-like B cells that can be subdivided into B1a and B1b cells. It was reported that B1a cells help suppress infection, inflammation, allergies, autoimmune diseases and cancer.^[27]^ In the area of cardiovascular diseases, B1a cells and marginal zone B cells produce IgM, an anti-oxidized low-density lipoproteins antibody, which reduces foam cell formation and resists atherosclerosis, thereby preventing cardiovascular events and MI.^[28–30]^ It has also been shown that B1a cells prevent the formation of vulnerable atherosclerotic plaques and MI by secreting interleukin 10 (IL-10).^[29,31]^ It can effectively control the inflammatory response and attenuate aseptic cardiac injury by regulating the activation state of mast cells and inhibiting the differentiation of Th1 and Th17 cells in aseptic cardiac injury and by reducing the secretion of pro-inflammatory cytokines from DCs.^[32]^ what‘s more, toll-like receptors may be involved in the anti-atherosclerotic activity of B1a cells.^[29]^ Karadimou et al injected Apoe/mice with advanced atherosclerotic lesions with Toll-like receptor 7 and 5 weeks after the experiment found an increase in the expansion of marginal zone B cells and Treg, as well as an increase in plasma IgM antibodies against oxidized low-density lipoproteins and a decrease in plasma cholesterol levels in spleen.^[33]^ In the last 2 years, a pilot study demonstrated that patients with acute myocardial infarction (AMI) had significantly lower levels of CD24hiCD38hi B-reg cells than patients with stable CAD by quantitatively comparing regulatory B cells in patients with ST-elevated MI to those with stable angina.^[34]^ Yue Xu et al found that increased B-cell counts were positively correlated with enhanced ejection fraction in MI patients undergoing percutaneous coronary intervention, and that injection of bone marrow naïve B-cells may play an important role in reducing post-ischemic injury, preventing adverse ventricular remodeling and improving cardiac function after acute MI.^[35]^ Even though a large number of studies have demonstrated a negative correlation between B cells and the development of MI, the initial studies of B cells on cardiovascular disease started with immune diseases such as systemic lupus erythematosus, where the pro-inflammatory role of B cells can exacerbate cardiovascular injury. Thus the role of B cells in cardiovascular disease remains an ambiguous state. In this study, we performed MR experiments based on publicly available GWAS data to further corroborate the beneficial effects of B cells on MI.

We are under the impression that T cells and macrophages are the primary cells infiltrating atherosclerotic plaques and ultimately causing ACS such as MI.^[36]^ Most plaque T cells are CD4 + TCRαβ+ T cells, which play a key pathogenic role in atherosclerosis,^[37]^ but as early as the 1970s, Gershon and Kondo discovered that T cells not only enhance the immune response but also suppress it.^[38]^ which was later named regulatory T cells (Tregs). Tregs achieve immunosuppression through 4 main mechanisms: (1) regulation of DC cells through the mechanism of CTLA-4 interaction with CD80 and CD86; (2) cell lysis mediated by granzymes and perforin proteins; (3) production of inhibitory cytokines such as IL-10, transforming growth factor β, and IL-35; and (4) metabolic destruction of T cells through the IL-2, CD39, and CD73 pathways causing metabolic disruption of effector T cells. Together, these mechanisms enable Tregs to effectively inhibit immune cell activity and maintain the body’s immune homeostasis.^[39]^ Treg can be divided into a number of subsets, including CD4 + CD25 + Foxp3 + cells, IL-10)producing type 1 regulatory T cells, transforming growth factor β-producing type 3 helper T cells, CD8 + regulatory T cells, natural killer T cells, CD4-CD8-T cells, and γδT cells.^[40]^ A large number of studies have demonstrated the protective effects of Treg on atherosclerosis and unstable plaques, the most representative of which is the CD4 + CD25 + Foxp3 + cells, which plays a role in preventing atherosclerosis and MI by down-regulating the inflammatory response.^[37,39,41,42]^ The second Treg that exerts a protective effect is CD8 + regulatory T cell, including CD8 + CD25 + T cells and Qa-1 restricted regulatory T cells. CD8 + CD25 + T cells function like CD4 + Tregs, it has been shown to reduce plaque size, decrease macrophage content and inhibit CD4 + T cell proliferation in mouse adoptive cell transfer assays,^[43]^ and it does the same in humans.^[44]^ Qa-1 is the murine homologue of human leukocyte antigen E, a non-classical major histocompatibility complex class Ib molecule that interacts with CD94/NKG2A receptors on CD8 + T cells, NK and NKT cells, thereby attenuating the activity of these cells. The interaction of CD8 + T cells with Qa-1 inhibits T follicle helper cell-mediated activation of germinal center B cells, thereby inhibiting lesion development.^[45]^ In our study, 5 immunophenotypes belonging to the Treg panel were associated with the development of MI, among them, CD28 + CD45RA- CD8dim T cell absolute count, CD127 on CD28 + CD45RA + CD8 + T cell, CD4 on CD39 + activated CD4 regulatory T cell and CD8 on CD28- CD8 + T cell, these 4 immunophenotypes promote MI. While the risk of MI decreased with an increase in the proportion of CD28 + CD45RA + CD8 + T cell. A pivotal feature of CD8 + Tregs is the low-level expression of the co-stimulatory molecule CD28 (CD28dim), and adoptive transfer of CD8 + CD28dim T cells can effectively diminish disease severity.^[46]^ CD45 or leukocyte common antigen is a family of all surface tyrosine phosphatases and is expressed on all types of leukocytes. Naïve T lymphocytes express the 205-220-kd form of leukocyte common antigen, CD45RA; T cells express the 180-kd form, CD45RO, after the first round of activation by specific antigens, Analysis of plaque lymphocytes by Sten Stemme et al showed that most plaque T cells are memory cells, i.e., express CD45RO.^[47]^ The phenotypes to which the CD28 + CD45RA- CD8dim T cell belongs are all contrary to previously reported protective phenotypes, so we hypothesized that it is a risk factor for the development of MI. Guillaume Churlaud et al found by flow cytometry that in mice, CD4 Treg and CD8 Treg are defined only by the co-expression of FOXP3 and CD25. In humans and, to a lesser extent, in mice, CD4 Treg and CD8 Treg are also characterized by lower levels of CD127 compared to effector T cell.^[44]^ In our study CD127 on CD28 + CD45RA + CD8 + T cells expressing high levels of CD127 promoted the development of MI, consistent with previous findings. CD39 is a cell surface enzyme that removes nucleotides by hydrolytic cleavage and is expressed on almost all Foxp3 suppressor cells. Giovanna Borsellino et al found by analyzing a population of multiple sclerosis that the activity of CD39 has 2 synergistic anti-inflammatory effects: the removal of ATP and the production of immunosuppressive nucleoside adenosine.^[48]^ Other studies have shown that CD39 mediates the cardioprotective effects of Treg mainly by promoting adenosine production, regulating Akt and ERK pathways, inhibiting cardiomyocyte apoptosis, and reducing neutrophil infiltration.^[49]^ Study by Janine van Duijn et al highlights the anti-inflammatory role of CD39 + CD8 + T cells in advanced atherosclerosis.^[50]^ All previous studies have demonstrated that CD4 on CD39 + activated CD4 regulatory T cell may play a protective role against MI, but our study suggests that it may be a risk factor for MI. Newer study suggests complete deletion of CD39 has anti-atherosclerotic effects in ApoE-deficient mice.^[51]^ This may explain our results, but further research is needed to confirm it. While CD28 + CD45RA + CD8 + T cell had a protective effect on MI highly consistent with previous studies.

Four immunophenotypes in the maturation stages of T-cell panel were associated with the occurrence of MI, of which 3, i.e., Terminally differentiated CD4 + T cell Count, Terminally differentiated CD8 + T cell, CD45RA on naive CD4 + T cell, were correlated positively with the occurrence of MI, and the only one, named Effector Memory CD4 − CD8 − T cell %T cell, was negatively related to the incidence of MI. Overall, the effect of T cells on MI is to promote its occurrence. It is common sense that as people age, their body functions will gradually deteriorate. Immunosenescence is strongly associated with morbidity in the elderly. Senescence is particularly evident in adaptive immune function, as evidenced by a decrease in naïve T cell production, accumulation of effector and terminally differentiated T cells, and a decline in antibody responses to antigenic stimuli in the elderly. Chronic, sterile, low-grade inflammation also develops with aging. Early data from the OCTO Longitudinal Study of Immunity showed that people in their eighties had a more advanced immune senescence characterized by a higher frequency of CD8 + lymphocytes than CD4 + lymphocytes.^[52]^ Gustavo Campos Ramos et al proposed that myocardial aging is mediated by T cells, particularly CD4 + T cells.^[53]^ Murilo Delgobo et al revealed the role of terminally differentiated T cells in promoting the inflammatory transition in the heart.^[54]^ The study by Enrico Ammirati et al indicated that effector memory T cells had a significant and direct correlation with total plasma cholesterol and LDL cholesterol, which were the most strongly associated T cell subset in the coronary vascular zone at different stages of atherosclerosis.^[55]^ In the light of the above studies, it is reasonable to believe that terminally differentiated CD4 + T cell Count, Terminally Differentiated CD8 + T cell, CD45RA on naive CD4 + T cell, and other mature stage T cells can promote the occurrence of MI. As for the effector memory CD4-CD8- T cell, we consider that it does not express the pathogenic phenotypes CD4 + T cell and CD8 + T cell, and thus inhibits the occurrence of MI.

Beyond the immunophenotypes described above, there are 7 other immunophenotypes belonging to the TBNK panel that are relevant to MI. Four of them, including absolute counts of human leukocyte antigen (HLA) DR++ monocytes, absolute counts of CD8dim natural killer T cells, absolute counts of B cells, and CD8 on CD8 + T cells, were positively correlated with the development of MI, whereas the other 3, that is, CD45 on CD4 + T cells, FSC on HLA DR + natural killer cells, and HLA DR on HLA DR + T cell, were negatively associated with the development of MI. Monocytes are implicated in the progression of cardiovascular disease in humans. They are divided into 3 main subpopulations: classical (cMo), intermediate (iMo), and non-classical (nMo). Of these, cMo and iMo have been shown to be directly related to cardiac dysfunction.^[56–60]^ The literature reports an increased frequency of iMo (iMo_HLADR + CXCR3 + CD206+) in patients with cardiovascular disease and that upregulation of iMo is associated with plaque rupture.^[61]^ Several studies have shown that natural killer T cells promote atherosclerosis formation and facilitate plaque rupture.^[62–65]^ In addition to the previously mentioned CD8 + Tregs, CD8 + T cells are involved in the formation of atherosclerosis and the development of MI in a perpetrator fashion. CD8 + T cells control monocytogenesis and macrophage accumulation in early atherosclerosis. In addition, CD8 + T cells exert cytotoxic effects in atherosclerotic plaques and lead to macrophage death and necrotic core formation. Most notably, local cytotoxic CD8 + T cell responses may trigger endothelial injury and plaque erosion in ACS.^[45]^ In these case, absolute counts of HLA DR++ monocytes, absolute counts of CD8dim natural killer T cells, absolute counts of B cells, and CD8 on CD8 + T cells as the risk factors of MI would make sense. As to why the increase in bsolute counts of B cells increased the risk of MI, and why CD45 on CD4 + T cells, FSC on HLA DR + natural killer cells, and HLA DR on HLA DR + T cells were negatively correlated with the occurrence of MI, we do not have a clear explanation.

DCs are an important subset of immune cells that play a crucial role in the immune system, predominantly responsible for capturing, processing and presenting antigens to stimulate the immune response of T cells and B cells.^[66]^ The only immunophenotype that was in the DC panel and was associated with the development of MI was FSC-A on granulocyte, which was in a promotive relationship in the occurrence of MI. Granulocytes (neutrophils, eosinophils, and basophils) are the most abundant circulating cells in the innate immune system,^[67]^ of which the most prominent granulocytes are neutrophils. Neutrophils, the most abundant leukocyte type in the human circulation, are also the predominant cell type during acute inflammatory responses. It is well known that neutrophils are often used as a marker to assess the degree of inflammation and that MI cannot occur without the dual effects of atherosclerosis and inflammation. It has been shown that neutrophils can trigger atherosclerosis by increasing endothelial cell permeability, accelerating foam cell formation, and promoting pro-inflammatory production by macrophages.^[68,69]^ In addition to this, neutrophils can communicate with the environment through proteins in the NET, a complex containing the DNA antimicrobial peptide-associated antimicrobial peptide, which induces the production of interferon-alpha by plasma cell-like DCs, leading to the development of atherosclerosis in atherosclerosis-susceptible mice.^[70,71]^ This pathway also leads to atherosclerosis and the formation of vulnerable plaques, further contributing to the development of MI. Regarding eosinophils, observational studies have speculated that eosinophils are involved in the inflammatory process of MI by comparing serum eosinophil cationic protein and peripheral blood eosinophil counts at the beginning of MI. In another study, by analyzing thrombus aspiration specimens in emergency coronary angiography, it was found that the thrombus of patients with MI contained a large number of eosinophils and had a large thrombus area, and it was considered that eosinophils promote the growth of coronary thrombus and lead to the development of MI.^[72]^ The effect of basophils on cardiovascular disease is poorly described in the literature with incompletely defined mechanisms. Studies only shown that basophils have a reparative effect on the infarcted myocardium after MI via the secretion of IL-4/IL-13,^[73–75]^ but more recent studies have shown that monocyte-derived macrophages and other leukocytes, such as DCs, mast cells, B cells, T-cells, NK-cells, and Treg-cells, also play an important role in the healing of MI. In summary, the effect of granulocytes on MI was promotive, which is compatible with our research findings.

Monocytes are an important component of the cardiac innate immune system and can be classified into 3 distinct subpopulations of monocytes based on the expression of CD14 and CD16, that is, classical monocytes (CD14 + CD16−), intermediate monocytes (CD14++ CD16+) and non-classical monocytes (CD14 − CD16+).^[76]^ In the early stages of atherosclerosis, inflammation induces the recruitment of circulating monocytes arteriolar lumen, which differentiate into macrophages and become the main cell population involved in atherosclerotic plaque formation.^[77]^ During myocardial ischemia, monocyte-derived macrophages exert a pro-inflammatory role, releasing inflammatory cytokines such as IL-1β, tumor necrosis factor-α, nitric oxide, and IL-6, undergoing extensive phagocytosis, and may also form vulnerable plaques by secreting protein hydrolases and recruiting infiltrating immune cells into the heart, causing tissue damage and resulting in MI.^[78]^ Previous studies have demonstrated that CD14 + CD16 + monocyte subsets can be used as a predictive model of severity in patients with ACSs.^[57]^ In our research, there are 2 immunophenotypes present in the Monocyte panel, CD40 on CD14 + CD16- monocyte and CX3CR1 on CD14- CD16 + monocyte. One of them promotes the development of MI (CX3CR1 on CD14 − CD16 + monocyte), while another reduces the risk of MI (CD40 on CD14 + CD16 − monocyte). CD40 is the co-stimulatory receptor for CD40 ligand (CD40L/CD154), a type I transmembrane glycoprotein belonging to the tumor necrosis factor receptor superfamily, which is constitutively expressed in a wide range of cell types, including monocytes, macrophages, platelets, DCs, as well as endothelial and vascular smooth muscle cells. CD40-CD40L interaction is the link between immunity, CD40-CD40L interacts as a nexus between immune, inflammatory and coagulation states and is overexpressed in patients with atherosclerosis-related diseases, such as stroke and CAD, and several studies have shown that inhibition of CD40 or CD40L significantly reduces atherosclerosis in hyperlipidaemic mice.^[79–81]^ This contradicts our findings. However, it has since been noted that individuals with CD40 deficiency always have concomitant T-cell and dendritic cell dysfunction.^[82]^ By comparing CD40 expression in patients with stable angina to patients with AMI was significantly decreased in the AMI group, suggesting that there may be immune system dysfunction in patients with AMI.^[83]^ It may account for the conclusions of the present experiment. CX3CR1, a classic 7-transmembrane G-protein-coupled receptor, is the sole receptor for neurotrophic chemokine, and their interaction mediates leukostasis under flow conditions.^[84]^ In the absence of CAD, neurotrophic chemokine is usually not expressed. It is only when CAD is present that neurotrophic chemokine is expressed and upregulated.^[76,85]^ A gene sequencing experiment based on the GENIC case–control study of cerebral infarction confirmed that CX3CL1 promotes atherosclerotic thrombosis by promoting monocyte adhesion.^[86]^ A single-cell RNA sequencing (scRNA-seq) analysis demonstrated that CX3CR1 on monocytes plays an important role in promoting plaque fibrous cap rupture and that it is significantly expressed in both classical (CD14 + CD16-) and non-classical (CD14- CD16+) monocytes, but more so in non-classical (CD14- CD16+) monocytes.^[87]^ This is consistent with our findings.

Myeloid cells (granulocytes, monocytes, macrophages, and DCs) are innate immune guards against infections and become the protagonists of cardiovascular disease in the context of hyperlipidemia, most of which are generated by hematopoietic stem cells (HSCs).^[88]^ Our findings suggest that hematopoietic stem cell absolute count and CD45 on lymphocyte are associated with MI in myeloid cells panel, where hematopoietic stem cell absolute count increase the risk of MI, whereas CD45 on lymphocyte decreases the risk of MI. Heyde et al^[89]^ found that stem cell proliferation accelerates somatic cell evolution and clonal expansion with driver mutations, and that stem cell proliferation in atherosclerosis accelerates clonal hematopoiesis; thus, increased hematopoietic stem cell proliferation is an important factor contributing to the link between cardiovascular disease and clonal hematopoiesis. Ziguang Song et al^[90]^ further determined that the proportion of hematopoietic stem cells in MI patient samples was significantly higher than in normal samples by scRNA-seq. In terms of the pathogenic mechanisms of HSCs on MI, we know that hypercholesterolemia is the most important modifiable risk factor for cardiovascular disease, and that this metabolism alters the inflammatory response by reprogramming the function of HSCs and subsequent myelopoiesis. It has been shown that hyperlipidemia induces an increase in IL-6 expression in bone marrow endothelial cells, which enhances the proliferation and mobilization of HSCs as well as their differentiation to the myeloid lineage,^[91–93]^ and myeloid-differentiated leukocytes then release pro-inflammatory cytokines and proteases, triggering localized inflammatory responses, and subsequently monocytes respond to a variety of chemokines to be recruited to the site of inflammation, forming lipid-rich foamy cells and ultimately forming plaques At the same time, cytokines and proteases further destabilize the plaque and promote the development of MI.^[94]^ Large cohort studies have confirmed that Tet methylcytosine dioxygenase 2 (Tet2) deficiency is a central driver of the processes leading to this sequence of events through clonal hematopoiesis.^[95,96]^ In summary, for hematopoietic stem cell absolute count, our findings are consistent with those of previous studies. Circulating endothelial progenitor cells are involved in endothelial regeneration and neovascularization in cardiovascular disease, and the number of EPCs in the human circulation is inversely related to atherosclerosis and cardiovascular risk, and ultimately independently predicts cardiovascular disease progression. Several studies have shown that CD45 partially contains EPCs,^[97–99]^ so that we can reasonably hypothesize that CD45 on lymphocyte is inversely related to the occurrence of MI, which is consistent with our findings.

**Figure 6.**
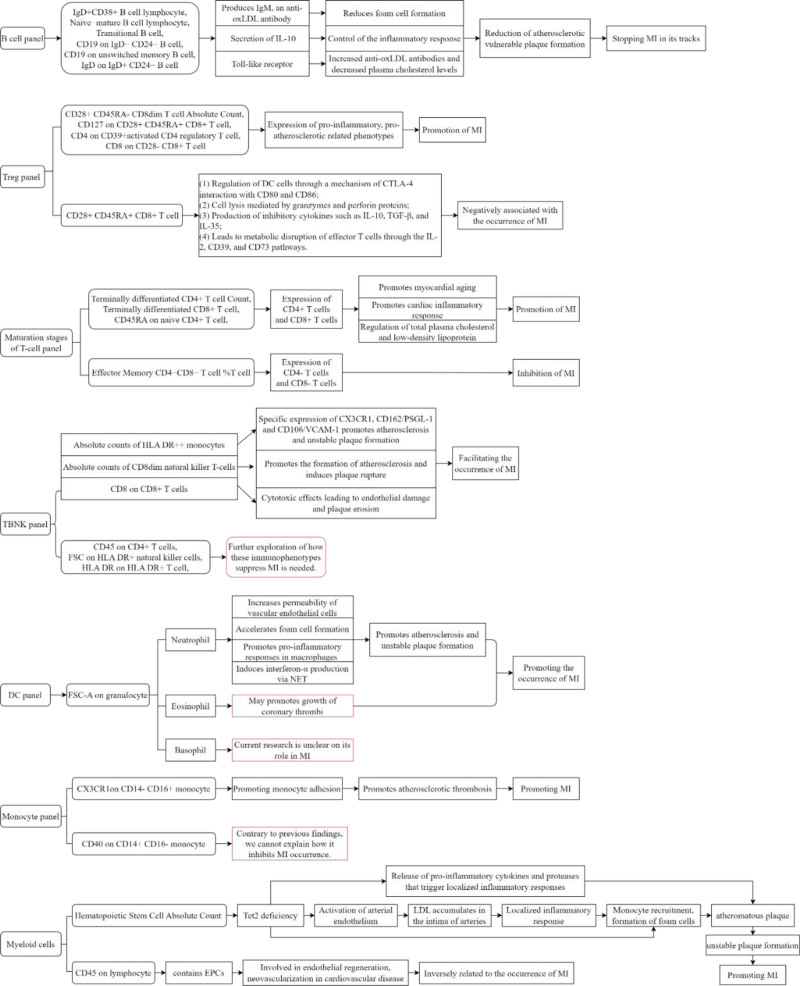
Mechanisms associated with the role of immune cell phenotypes on MI. MI = myocardial infarction.

In this study, a two-sample MR analysis was used, and the findings were based on GIVs, and causal inferences were made using a variety of robust MR analysis techniques, independent of horizontal pleiotropy and other variables. The discussion section of this paper provides an exhaustive overview of the impact of each outcome immunophenotype on MI. The possible pathways between various immune cells and MI are shown in Figure 6. Compared with previous studies, we provide more immune cell phenotypes that are causally related to MI, but our individual findings seem to contradict previous observations, and we speculate that there are 2 reasons for this contradiction: (1) observational studies are prone to reverse causation, and (2) from the perspective of the gene segments, the functions of the immune phenotypes expressed by different gene segments may be different.

Limitation: (1) this study relied on a European database, which limits the generalization of the results to other ethnic groups. Considering the global burden of MI, in order to enhance the reliability of the findings, the veracity of the inferred causality has to be confirmed in different populations, and therefore, careful data analysis and comparisons between populations of different races are needed. However, the current database of non-European populations is not complete, and we are unable to address this issue for the time being, considering the scientific nature and reliability of scientific research. It is expected that further improvement of the database or the emergence of more robust research methods in the future will allow us to carry on and develop our findings. (2) Publicly available GWAS data on immune cell phenotypes and MI lack the incorporation of information on specific characteristics such as age, sex, body weight, and degree of MI, nuances that can lead to the influence of confounding variables on the results. It is important to recognize that summary data may be subject to selectivity bias affecting selection and thus the generalizability of results. (3) The study’s flexible outcome assessment criteria may lead to more false positives, in our study, we were unable to find eligible SNPs with the threshold set at *P* < 5 × 10^-8^, so we chose the less stringent *P* < 1 × 10^-5^, which, it must be admitted, may have led to the appearance of false-positive results to a certain extent, but in the discussion section, we further elaborated on the effect of the resultant immune cell phenotype on MI in the context of the clinical study, and our results were mostly consistent with those of previous studies, which further confirmed the reliability of our study. (4) In addition, we only demonstrated a causal relationship between the resultant immunophenotype and MI in this study, and we were not able to assess the magnitude of the causal effect value, In short, we only verified our opinion in terms of nature and did not quantify it. Moreover, due to the limitation of funds and test equipment, we could not conduct a clinical trial to further confirm our results, and we hope to have the opportunity to conduct further experiments in the future to confirm this.

## 5. Conclusion

We used MR analysis to reveal the causal relationship between various immune phenotypes and MI, revealing the intricate relationship between MI and the immune system. Our results indicated that higher B cells and Treg were negatively correlated with the occurrence of MI, whereas mature stage T cells, monocytes, and Myeloid cells were mostly positively correlated with the occurrence of MI. The implications of our study for clinical practice can be summarized as follows. MI is a serious life-threatening disease, and immune cells are present throughout the development of MI; however, not all immune cells contribute to MI, and in order to determine their efficacy, large-scale randomized controlled trials are necessary to validate the potential of novel therapies that aim to protect B cells and regulatory T cells and to inhibit maturation-phase T cells, monocytes, and myeloid cells. myelin-like cells, among other novel therapies. With the continuous development of technology, we can use single-cell scRNA-seq can explore gene expression profiles and predict drugs through protein-protein interactions and molecular docking, for example, it has been found that NLRP3 inhibitors show stronger atheroprotective activity against atherosclerosis in chimeric mice recombinantly reconstituted with Tet methylcytosine dioxygenase 2-deficient cells, the DB05490 may act as a potential inhibitor of hematopoietic stem cell-associated genes, and both drugs may be used as therapeutic agents for MI. This study provides more insights into immune cell phenotypes as genetic markers of MI, This will start a new chapter in the prevention and treatment of MI from an immune perspective. In addition, our study successfully reduced the effects of reverse causality, other variables, and other unavoidable confounding factors.

## Acknowledgments

The author is very grateful to all those who participated in the genomics analyses used in this manuscript and to the researchers who made the data from these genomics analyses publicly available. I would also like to thank all my teachers and friends who gave me valuable advice and suggestions during the course of this study, as well as myself for taking each step seriously and finalizing this study.

## Author contributions

**Data curation:** Yinyin Xu.

**Formal analysis:** Yinyin Xu.

**Methodology:** Jing Yang.

**Validation:** Yanhua Zhang, Rong Xue, Guojiang Zhang.

**Writing – original draft:** Yinyin Xu.

**Writing – review & editing:** Yanhua Zhang, Guojiang Zhang.
